# *In vivo* evolution of resistance to contemporary β-lactam/β-lactamase inhibitor combinations during treatment of a KPC-producing *Serratia marcescens* infection

**DOI:** 10.1128/aac.01651-25

**Published:** 2026-03-05

**Authors:** Daniel C. Bailey, Salvador Castañeda-Barba, Katie E. Barry, Puthayalai Treerat, Matthew A. Crawford, Molly A. Hughes, Amy J. Mathers

**Affiliations:** 1 Division of Infectious Diseases and International Health, Department of Medicine, University of Virginia School of Medicine 214842 , Charlottesville, Virginia, USA; 2 Clinical Microbiology Laboratory, University of Virginia Health System 12350, Charlottesville, Virginia, USA; 3 Department of Pathology, University of Virginia School of Medicine 189557, Charlottesville, Virginia, USA; Entasis, Big Bay, Michigan, USA

**Keywords:** *Klebsiella pneumoniae *carbapenemase, KPC-44, *Serratia marcescens*, carbapenem-resistant *Enterobacterales*, ceftazidime-avibactam, allele variation, multidrug resistance, carbapenem resistance, avibactam resistance, vaborbactam resistance, relebactam resistance

## Abstract

*Klebsiella pneumoniae* carbapenemases (KPCs) are a family of serine β-lactamases that confer broad antibiotic resistance by hydrolyzing virtually all β-lactam (BL) agents. Contemporary β-lactamase inhibitors (BLIs) such as avibactam were developed to neutralize the activity of KPCs and other clinically important carbapenemases. Ceftazidime-avibactam (CZA), a BL/BLI combination in which the cephalosporin ceftazidime is protected from KPC-mediated hydrolysis, demonstrated improved outcomes in early clinical use. However, CZA-resistant isolates soon emerged. Herein, we describe a challenging clinical case in which high-level resistance to CZA evolved during therapy for a complicated infection caused by carbapenem-resistant *Serratia marcescens* harboring *bla*_KPC-2_. Whole-genome sequencing, analysis of antibiotic resistance genes, and phenotypic susceptibility assays of serial *S. marcescens* isolates revealed that resistance arose via a 45-nucleotide in-frame duplication within *bla*_KPC-2_, yielding KPC variant 44 (*bla*_KPC-44_). Notably, the evolution of *bla*_KPC-44_ not only conferred resistance to CZA but also marked cross-resistance to meropenem-vaborbactam and imipenem-relebactam while remaining susceptible to cefiderocol. To our knowledge, this report represents the first description of *bla*_KPC-44_ emerging outside of *K. pneumoniae* and among the few documenting a CZA-resistance-conferring KPC variant emerging in a non*-K*. *pneumoniae Enterobacterales* species. Ultimately, the evolved strain persisted despite therapy throughout a fatal clinical course, underscoring the potential for CZA selective pressure to drive treatment-emergent resistance to multiple contemporary BL/BLI agents.

## INTRODUCTION

The emergence and spread of carbapenem-resistant *Enterobacterales* (CRE) represents a major global health challenge, as carbapenems are among the most effective “last-resort” β-lactam (BL) antibiotics typically reserved for treating severe infections caused by multidrug-resistant (MDR) gram-negative bacteria (1). Carbapenem resistance is frequently mediated by carbapenemases—a diverse group of β-lactamase enzymes capable of inactivating nearly all currently available BL antibiotics. First identified in 1996 (2), *Klebsiella pneumoniae* carbapenemases (KPC) are serine (non-metallo) β-lactamases (Ambler class A) that have since disseminated globally among gram-negative pathogens and are now endemic in the United States and elsewhere (3). Infections caused by CRE have historically lacked effective treatment regimens and are associated with three- to fourfold higher mortality than those caused by carbapenem-susceptible organisms (4, 5). In response to these challenges, novel β-lactamase inhibitors (BLIs) were developed to inhibit KPCs and other clinically important carbapenemases, thereby restoring the activity of paired BL antibiotics (6).

Ceftazidime-avibactam (CZA), the first combination agent to incorporate a novel diazabicyclooctane BLI with activity against KPC-producing organisms, was approved for clinical use in 2015. Its authorization was based on clinical trials demonstrating noninferiority to carbapenems for treating a variety of infections caused by carbapenem-susceptible organisms (7–9). However, these trials did not include the primary target organisms for which CZA would ultimately be used (i.e., carbapenemase-producing organisms), leaving significant gaps in understanding the drug’s efficacy and durability against its intended targets. Despite these uncertainties, early post-marketing studies encouragingly demonstrated improved clinical outcomes when CZA was used to treat invasive infections caused by carbapenem-resistant organisms (10, 11).

While optimism stemmed from the ability to directly inhibit these resistance-conferring enzymes and avoid reliance on highly toxic, less efficacious agents such as colistin (12), KPC-2 variants resistant to avibactam were observed in laboratory studies even before clinical introduction (13). Consistent with these findings, CZA-resistant isolates soon began to emerge in clinical settings. Multiple studies have since shown that resistance to CZA frequently arises from mutations in circulating *bla*_KPC-2_ and *bla*_KPC-3_ alleles. The capacity of KPCs to evolve such resistance-conferring variants has been regarded as both impressive and alarming (14, 15).

Herein, we describe a challenging clinical case in which CZA was used to treat a complicated infection caused by carbapenem-resistant (*bla*_KPC-2_-harboring) *Serratia marcescens*, a less common *Enterobacterales* species that was underrepresented in initial CZA clinical trials. During therapy, high-level resistance to CZA emerged, accompanied by cross-resistance to meropenem-vaborbactam (MVB) and imipenem-relebactam (I-R). The overarching goal of this study is to use real-world experience to further our understanding of how MDR bacteria evade the latest BL/BLI agents in order to better inform their optimal clinical use and preserve their therapeutic utility into the future.

## MATERIALS AND METHODS

### Clinical isolate identification and antimicrobial susceptibility testing

*S. marcescens* clinical isolates were processed and analyzed by the Clinical Microbiology Laboratory at the UVA Health University Medical Center (Charlottesville, VA, USA) in the context of clinical care. Rapid molecular identification of positive blood cultures was carried out by multiplex PCR (cobas ePlex BCID, Roche Diagnostics, Indianapolis, IN, USA). Species identification was confirmed using matrix-assisted laser desorption ionization-time of flight mass spectrometry (Vitek MS Prime, bioMérieux, Durham, NC, USA). *In vitro* AST to determine minimum inhibitory concentrations (MICs) was carried out using the Sensititre platform (Thermo Fisher Scientific, Waltham, MA, USA) utilizing their gram-negative MIC plates (GN6F and RGNX2F) and interpreted according to Clinical and Laboratory Standards Institute (CLSI) guidelines available at the time (16).

MIC testing with cefiderocol was initially performed on the Sensititre platform, but results were not reported after the manufacturer recalled the testing plate due to inaccurate results (17). Isolates were later manually retested via broth microdilution (BMD) in iron-depleted media according to current CLSI guidance (18). Iron-depleted cation-adjusted Mueller Hinton broth (ID-CAMHB) was prepared by treating rehydrated BBL CAMHB (Becton Dickinson, Sparks, MD, USA) with cation-exchange Chelex 100 resin (100–200 mesh particle size, sodium form) for 6 h at ambient temperature. After filtration of resin, CaCl_2_, MgCl_2_, and ZnCl_2_ were added to achieve the final cation concentrations specified by CLSI guidance (20 mg/L Ca^2+^, 10 mg/L Mg^2+^, and 0.5 mg/L Zn^2+^). The concentration of iron in the final media was confirmed to be ≤0.03 mg/L using the VISOCOLOR HE kit (MACHEREY-NAGEL, Duren, Germany). Cefiderocol was purchased from BOC Sciences (Shirley, NY, USA) and reconstituted in sterile saline before being diluted to 2× working concentrations in ID-CAMHB. MIC determination was based explicitly on European Committee on Antimicrobial Susceptibility Testing BMD reading guidelines for cefiderocol (19). All strains were tested in triplicate. Each batch of testing included the quality control strains *Pseudomonas aeruginosa* ATCC 27853 and *Escherichia coli* ATCC 25922 to confirm satisfactory assay performance (i.e., MICs against these strains were always within the acceptable range of 0.06–0.5 µg/mL) (18).

### Whole-genome sequencing, antibiotic resistance gene profiling, and clonality analysis

A single colony of each *S. marcescens* colony morphotype was freshly subcultured onto Sheep’s Blood agar (Remel, Thermo Scientific, Lenexa, KS, USA). Each *S. marcescens* isolate was designated with a unique internal sequencing identifier: *Sm*-1: CAVp640; *Sm*-2: CAVp652; *Sm*-3a: CAVp655; *Sm*-3b: CAVp656; *Sm*-4a: CAVp657; and *Sm*-4b: CAVp653. For short-read sequencing, DNA was extracted from the bacterial colonies using the Qiagen EZ1 DNA Tissue Kit (Qiagen, Hilden, Germany) per the manufacturer’s instructions and checked for acceptable quality and quantity using a Qubit 4 fluorimeter (Thermo Fisher Scientific). Sequencing libraries were prepared using the Illumina DNA Prep Kit (Illumina, Inc., San Diego, CA, USA) following the manufacturer’s protocol. Paired-end sequencing was carried out using a MiSeq v2 300-cycle Reagent Kit (Illumina) on an Illumina MiSeq platform. For long-read sequencing, DNA was extracted from bacteria using a Qiagen Genomic DNA Kit (100/G tips, QIAGEN) per the manufacturer’s protocol. For all isolates besides *Sm*-3b, library preparation was performed using the Oxford Nanopore Technologies (ONT) Ligation Sequencing Kit (SQK-LSK109, Oxford Nanopore Technologies, Oxford, UK) with the Native Barcode Kit (EXP-NBD104), followed by sequencing on an ONT MinION instrument using R9.4.1 flow cells. Due to difficulties with assembly of *Sm*-3b sequences, this isolate was re-analyzed using an ONT GridION instrument (equipped with R10.4.1 flow cells) following library preparation with an ONT Rapid Barcode Kit (SQK-RBK114.24).

The raw Illumina paired-end reads for each isolate were quality filtered to remove Illumina adapter sequences and trim low-quality tail ends using *TrimGalore* (20). Variant calling was then performed using the *Snippy* (v4.6.9) pipeline (21), by mapping trimmed reads to the *S. marcescens* isolate CAV1761 reference genome (22). Single-nucleotide variants (SNVs) were extracted from the *Snippy* core genome output using *SNP-sites* (v2.5.1) (23). *SNP-dists* (v0.8.2) was then used to convert the FASTA alignment to a pairwise SNV distance matrix for downstream clonality analysis (24).

For ONT long-read data, *Dorado* (v.0.6.0) was utilized for base calling (25). Sequencing data from isolate *Sm*-3b were analyzed using model dna_r10.4.1_e8.2_400bps_sup (v4.3.0), while all other isolates were analyzed using model dna_r9.4.1_e8_sup (v3.6). Hybrid assemblies were generated using *Hybracter* (v0.11.2) (26). Default parameters for the “hybracter hybrid” command were used, including automatic genome size estimation with the “-- auto” flag. Assembled genomes were annotated for ARGs using *AMRFinder* (v4.0.23, database v2025-07-16.1) with default parameters (27, 28). Contigs were annotated with plasmid replicons using the latest version of *PlasmidFinder* (v2.1.6) and its accompanying database (v2.2.0) (29).

## RESULTS

### Clinical case description

**Fig 1:**
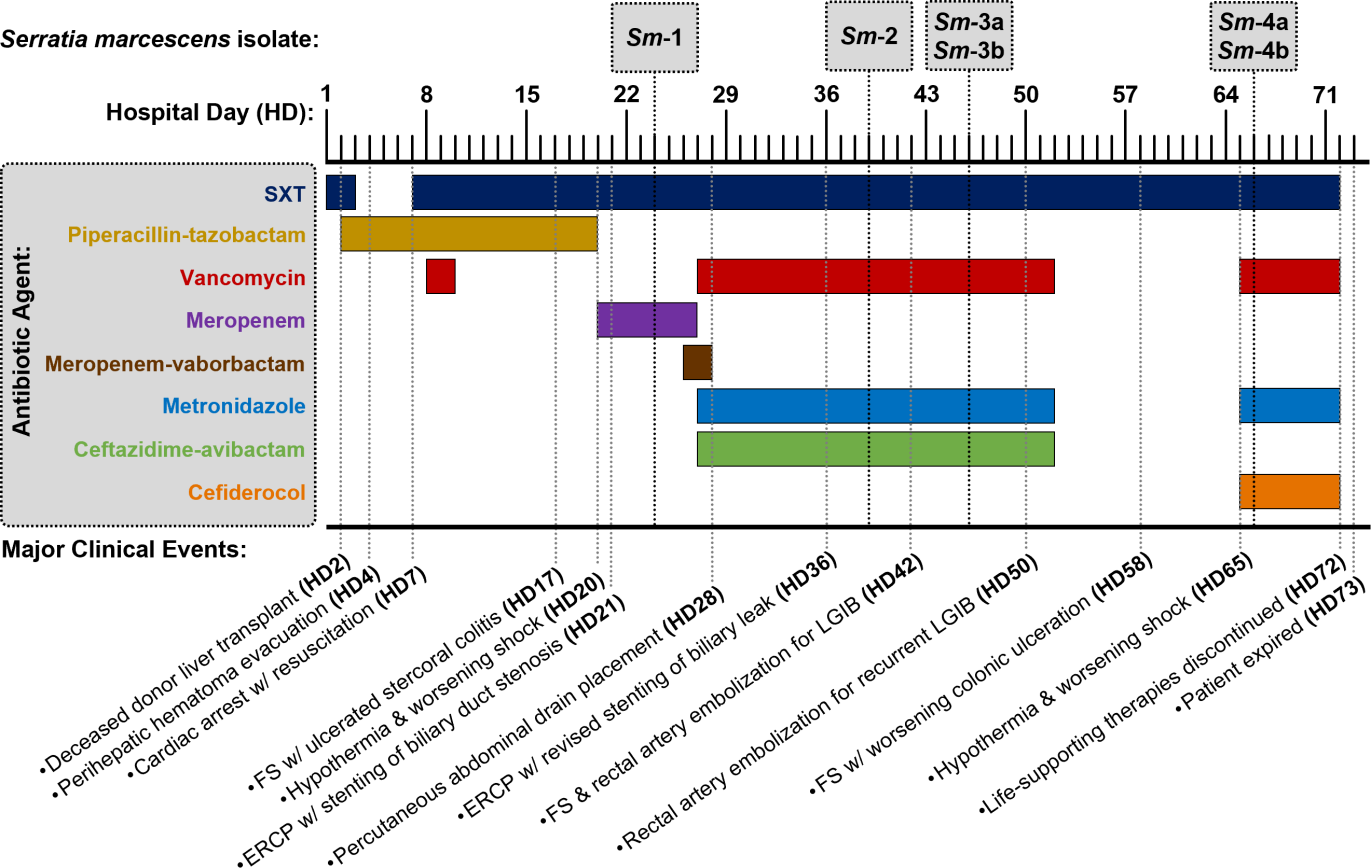
Clinical timeline summarizing antibiotic treatment periods, *Serratia marcescens* (*Sm*) isolate collection timing, and major clinical events. ERCP, endoscopic retrograde cholangiopancreatography; FS, flexible sigmoidoscopy; LGIB, lower gastrointestinal bleeding; SXT, trimethoprim–sulfamethoxazole.

A patient in their 50s with decompensated hepatic cirrhosis secondary to metabolic dysfunction-associated steatohepatitis was admitted to the UVA Health University Medical Center in 2021 with worsening acute-on-chronic liver disease (Fig. 1). Acute infections were ruled out, and the patient underwent orthotopic liver transplant on the second day of hospitalization (HD2). Per institutional perioperative liver transplant protocol, the patient received piperacillin-tazobactam (TZP) for surgical prophylaxis, and trimethoprim-sulfamethoxazole (SXT) was initiated on HD7 for standard opportunistic infection prevention. The early post-operative period was complicated by a large perihepatic hematoma prompting re-operation for evacuation, cardiac arrest, multifactorial shock treated with continuous vasopressors, oliguric kidney failure requiring renal replacement therapy (RRT), high-volume pleural effusions, respiratory failure necessitating mechanical ventilation via tracheostomy, and persistent lower gastrointestinal bleeding refractory to multiple endoscopic and endovascular interventions.

On HD20, the patient developed signs of sepsis characterized by hypothermia and worsening shock, though initial blood cultures were negative. Post-transplant immunosuppressive therapy (i.e., corticosteroids, tacrolimus, and mycophenolate mofetil) had been reduced due to concerns for emerging infection. Given a remote history of spontaneous bacterial peritonitis due to TZP-resistant *E. coli*, empiric antibiotic therapy for abdominal infection was transitioned to meropenem. Imaging revealed biliary duct stenosis with associated leak and intra-abdominal fluid collections. Cultures obtained on HD24-25 from blood, endotracheal sputum, and peritoneal abscess fluid grew *bla*_KPC_-positive *S. marcescens* (*Sm*-1), while *Enterococcus faecalis* (ampicillin and vancomycin susceptible) was also isolated from blood. Per institutional preference for treating KPC-producing organisms, antibiotic therapy was transitioned to include MVB on HD26. Subsequent susceptibility testing revealed borderline susceptibility to MVB (MIC 4 µg/mL) compared to CZA (MIC ≤ 2 µg/mL), prompting a switch to CZA on HD27.

The antibiotic regimen of CZA, vancomycin, and metronidazole was continued for 26 days. Combined antimicrobial therapy and source control via percutaneous drainage of intra-abdominal fluid collections and endoscopic retrograde cholangiopancreatography (ERCP) with biliary stenting resulted in resolution of sepsis and steady clinical improvement over approximately 3 weeks. In response to fluctuations in the patient’s cardiopulmonary status, surveillance sputum cultures from deep tracheostomy suctioning were obtained on HD39 (isolate *Sm*-2) and HD46 (isolates *Sm*-3a and 3b). These sputum specimens showed persistence of carbapenem-resistant *S. marcescens*, but ongoing recovery of this organism did not correlate with clinical or radiographic evidence of pneumonia. Interval development of CZA resistance in *Sm*-3 isolates further supported colonization rather than active infection. Consequently, broad-spectrum antibiotic therapy was discontinued on HD53, and the patient received only prophylactic SXT for 13 days without clinical deterioration.

On HD65, the patient again exhibited sepsis with hypothermia and worsening shock, prompting repeat infectious work-up and reinitiation of broad-spectrum antibiotics. Given the development of resistance to the latest generation BL/BLIs in the preceding *Sm*-3 sputum isolates, empiric therapy included cefiderocol, vancomycin, and metronidazole. Blood cultures were negative, but culture of bronchoalveolar lavage (BAL) fluid obtained on HD66 again grew carbapenem and CZA-resistant *S. marcescens* (isolates *Sm*-4a and 4b). After ongoing severe complications, the patient and family elected to withdraw life-sustaining therapies, and the patient passed on HD73.

### AST and genomic analysis of longitudinally collected *S. marcescens* isolates

**Fig 2:**
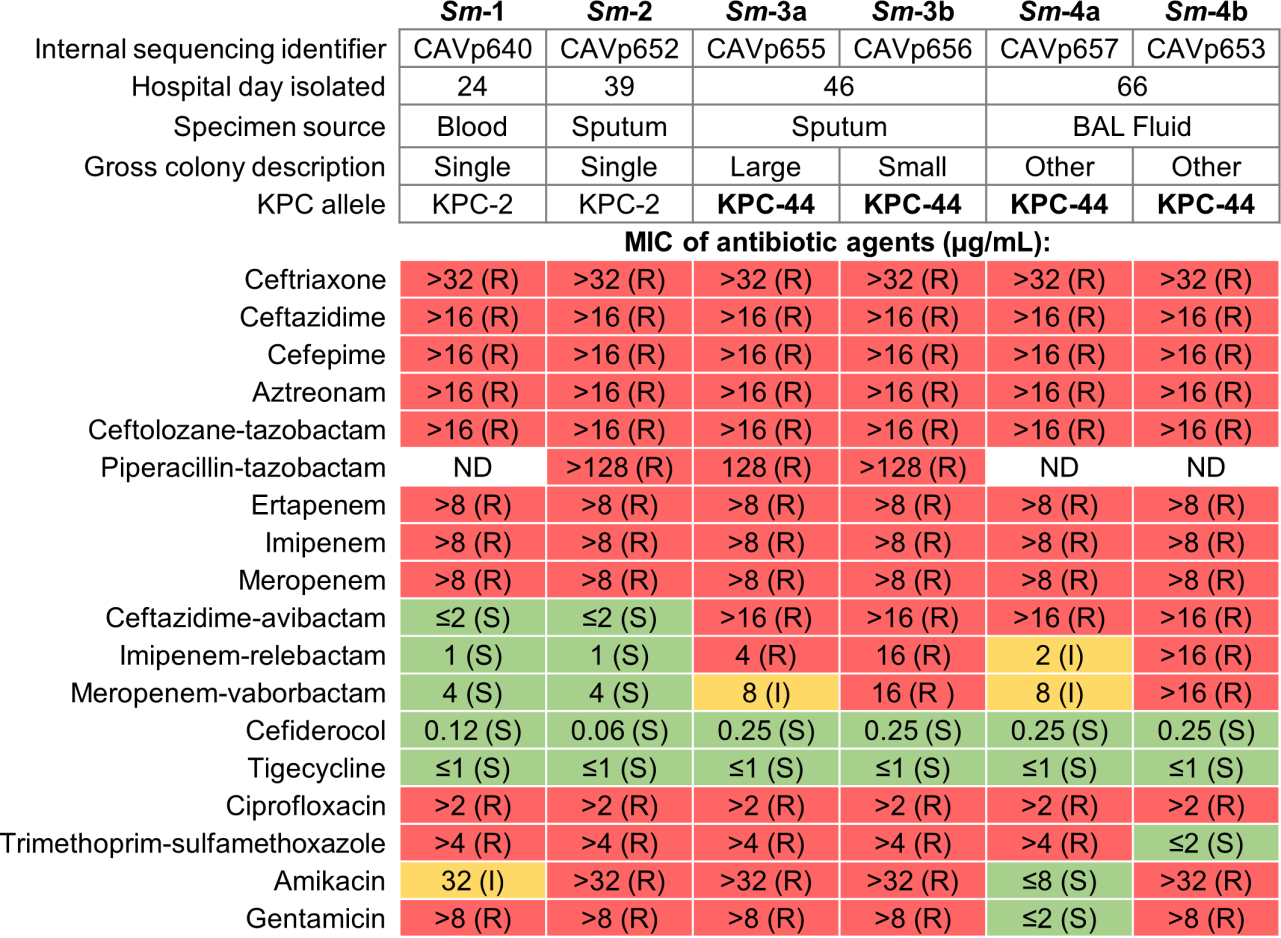
Summary of *Serratia marcescens* (*Sm*) isolate characteristics and antimicrobial susceptibility testing (AST) determined by minimum inhibitory concentration (MIC). ND, not determined; I, intermediate; R, resistant; S, susceptible; interpretations based on Clinical and Laboratory Standards Institute (CLSI) breakpoints.

**Fig 3:**
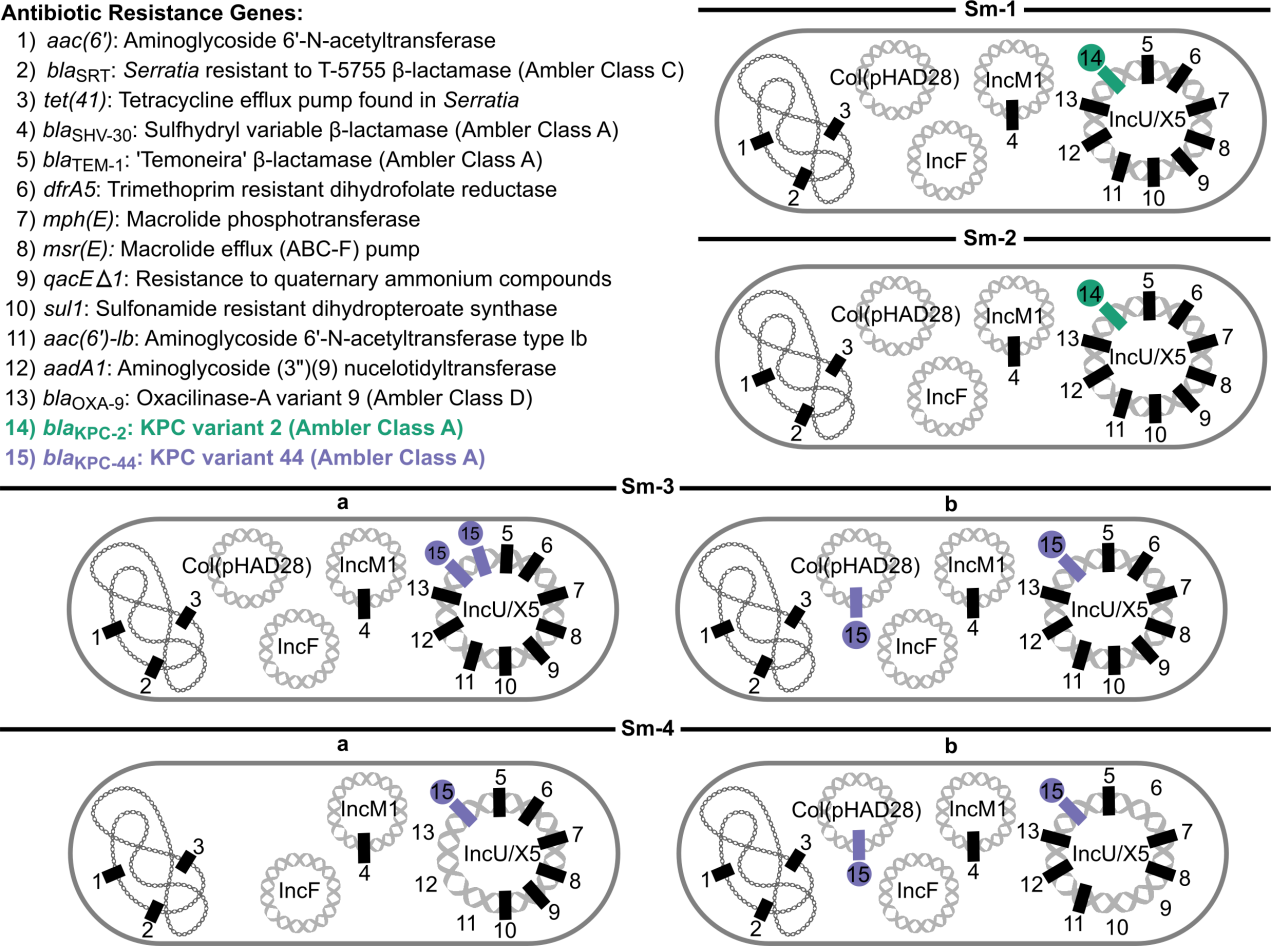
Summary of antibiotic resistance genes (ARGs) detected in longitudinally collected *S. marcescens* isolates *Sm*-1 through *Sm*-4. The bacterial chromosome and distinct plasmids harbored by each isolate are depicted. The presence of a given gene is indicated by a rectangle. The *bla*_KPC-2_ and *bla*_KPC-44_ alleles are highlighted in green and purple, respectively. The ordering of ARGs on each genetic element was chosen for visual clarity and does not reflect actual genetic structure.

Six isolates of *S. marcescens* were obtained from four clinical specimens collected on hospital days 24, 39, 46, and 66 (Fig. 1). Strains were designated *Sm-*1 through *Sm*-4 chronologically, with colony morphotypes denoted “a/b” when applicable. Specimens from HD46 and HD66 each yielded two grossly distinct colony morphologies, which were analyzed separately as individual strains. All six isolates underwent both *in vitro* AST (Fig. 2) and whole genome sequencing (WGS) (Fig. 3).

The first isolate, *Sm*-1, was recovered following the patient’s initial sepsis episode. In addition to being cultivated from blood, phenotypically indistinguishable *S. marcescens* were also obtained from endotracheal sputum and peritoneal abscess fluid cultures, consistent with a severe, disseminated infection likely originating as a complicated intra-abdominal infection (cIAI). AST of isolate *Sm*-1 revealed carbapenem resistance, as well as resistance to agents from multiple antibiotic classes including macrolides, aminoglycosides, fluoroquinolones, and SXT (Fig. 2). WGS identified 14 discrete ARGs concordant with phenotypic susceptibility (Fig. 3). Chromosomal ARGs included *tet41* (tetracycline efflux transporter), *aac(6*′) (aminoglycoside 6′-*N*-acetyltransferase), and *bla*_SRT_ (“*Serratia* resistant to T-5755” Ambler class C β-lactamase). Plasmid-encoded ARGs included *bla*_SHV-30_ (“sulfhydryl variable” Ambler class A β-lactamase) on IncM1, and 10 ARGs, including *bla*_KPC-2_, on IncU/M5. Plasmids Col(pHAD28) and IncF lacked putative ARGs. Phylogenetic analysis revealed that *Sm*-1 differed by only 16 SNVs compared to a *bla*_KPC_-harboring *S. marcescens* strain isolated at our institution in 2011 (30), and was closely related to other patient- and hospital-derived isolates (31–33). Taken together, these findings suggest infection with an endemic, nosocomial MDR *S. marcescens* strain.

The patient was treated with a multidrug regimen that included CZA, to which *Sm*-1 was quite susceptible (MIC ≤ 2 µg/mL). Accordingly, the patient responded favorably, and clinical improvement was observed. After 12 days of treatment, a surveillance endotracheal aspirate obtained on HD39 yielded *S. marcescens* (*Sm*-2) that remained CZA-susceptible. *Sm*-2 was genomically indistinguishable from *Sm*-1, with no detected SNVs, identical ARG content, and essentially identical AST results (Fig. 2 and 3).

A subsequent surveillance endotracheal sputum aspirate obtained on HD46, after 19 days of CZA therapy, again yielded *S. marcescens* (isolates *Sm*-3a and 3b). In contrast to preceding isolates, both displayed high-level CZA resistance, with MICs increasing from ≤2 to >16 µg/mL (Fig. 2). Moreover, this newly acquired CZA resistance was accompanied by concomitant resistance to MVB and I-R. Thus, within 12–19 days of *in vivo* CZA exposure, *S. marcescens* evolved resistance to three of the most advanced BL/BLI combinations in current clinical use, while maintaining carbapenem resistance.

**Fig 4:**
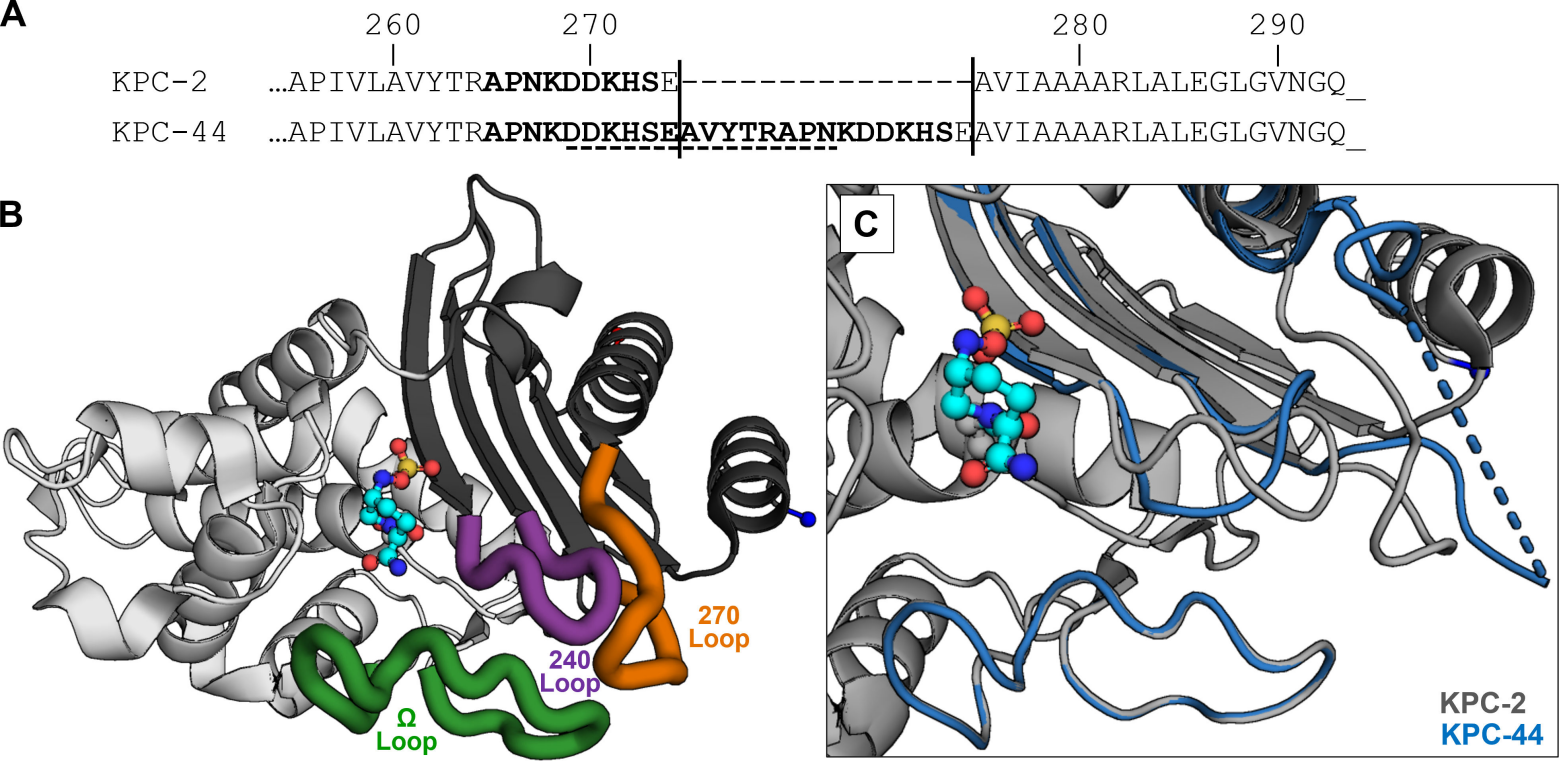
(**A**) Alignment of the *C*-terminal amino acid sequences of KPC-2 and KPC-44. The 15-amino acid duplication is delimited by vertical lines; the 270-loop regions are shown in bold, and disordered residues in the KPC-44 crystal structure are underlined with a dashed line. (**B**) Overall architecture of KPC-2 (PDB ID: 4ZBE) with carbamyl-avibactam (cyan carbon atoms) covalently linked to active-site nucleophile Ser70. The two enzyme subdomains are distinguished in light and dark gray. “Hot spots” for KPC mutations leading to CZA resistance include the Ω-loop (green), 240-loop (purple), and 270-loop (orange). (**C**) Close-up comparison of the “hot spot” loops and neighboring active site of KPC-2 (gray) overlaid with corresponding regions of KPC-44 (blue) (PDB ID: 8TMR) illustrating the substantial conformational changes induced by the 15-amino acid insertion. Fourteen unresolved residues in the 270-loop of KPC-44 are indicated by a dashed line. PDB, Protein Data Bank.

To investigate the mechanism of resistance, we performed genomic comparisons of sequential isolates. Pairwise SNV analysis across the six isolates revealed only 0–2 SNVs in the core genome. Given an estimated *S. marcescens* genomic mutation rate of 2.2 SNVs/genome/year (34), this is consistent with clonal relatedness. Similarly, the complement of 14 putative ARGs also remained constant in sequential isolates *Sm*-1 through *Sm*-3. However, a detailed analysis of *bla*_KPC_ alleles revealed a 45-nucleotide in-frame duplication within *bla*_KPC-2_, producing a 15-amino acid duplication (260–274) appended after position 274 in the *C*-terminal 270-loop of the KPC enzyme (Fig. 4A). This *bla*_KPC-2_ variant, associated with CZA resistance, was identified as KPC variant 44 (*bla*_KPC-44_) (35). Taken together, these data indicate that high-level resistance to CZA, MVB, and I-R arose through clonal evolution of the infecting lineage rather than replacement by a distinct strain harboring *bla*_KPC-44_.

The *Sm*-3 sputum culture yielded two distinct colony morphologies: *Sm*-3a (large) and *Sm*-3b (small). Genomic analysis confirmed subtle but notable differences. *Sm*-3a carried two *bla*_KPC-44_ copies on the IncU/M5 plasmid, whereas *Sm*-3b harbored two *bla*_KPC-44_ copies across distinct plasmids—one on the IncU/X5 plasmid and another on Col(pHAD28) (Fig. 3). Corresponding AST differences were modest, with *Sm*-3b exhibiting a twofold higher MIC against MVB and fourfold higher MIC against I-R compared with *Sm*-3a (Fig. 2). These findings suggest the emergence of distinct but closely related subpopulations under selective pressure.

After a 13-day antibiotic-free interval during clinical stabilization, sepsis recurred on HD65. Culture of BAL fluid obtained on HD66 again yielded two carbapenem-resistant *S. marcescens* isolates (*Sm*-4a and 4b). These isolates showed more pronounced genomic and phenotypic divergence. *Sm*-4a lost four ARGs (one of two *bla*_KPC-44_ copies, *aac(6*′*)-1b*, *aadA1*, and *bla*_OXA-9_) from the IncU/X5 plasmid and no longer carried the Col(pHAD28) plasmid (Fig. 3). These genetic changes corresponded with susceptibility to aminoglycosides and a twofold reduction in the I-R MIC (from 4 to 2 µg/mL) (Fig. 2). In contrast, *Sm*-4b exhibited SXT susceptibility associated with the loss of *sul1* (sulfonamide-resistant dihydropteroate synthase) and *dfrA5* (trimethoprim-resistant dihydrofolate reductase) from the IncU/X5 plasmid. Despite discontinuation of CZA and the initiation of a cefiderocol-based multidrug regimen, both *Sm*-4 isolates retained *bla*_KPC-44_ and high-level resistance to CZA. These observations suggest the evolution of increasingly divergent subpopulations under dynamic antibiotic pressure.

## DISCUSSION

Following liver transplantation, the recipient developed a nosocomial carbapenem-resistant *S. marcescens* cIAI with secondary bacteremia and persistent pulmonary colonization. Although intra-abdominal fluid collections were promptly drained and bacteremia rapidly cleared, the *S. marcescens* persisted within the patient’s respiratory tract throughout a 26-day course of CZA. Serial isolates demonstrated >8-fold increase in CZA MICs (Fig. 2), coinciding with mutation of *bla*_KPC-2_ to *bla*_KPC-44_ (Fig. 4). First described in 2018 in an MDR *K. pneumoniae* isolate, *bla*_KPC-44_ emerged after 34 days of CZA exposure in a patient colonized with *bla*_KPC-2_ (36). That patient recovered on a multidrug regimen including colistin, an option unavailable in this case due to the intrinsic polymyxin resistance of *S. marcescens* (37). Despite differences in species, timing, and infection type, both cases highlight how carriage of *bla*_KPC-2_-harboring CRE, prolonged hospitalization, and sustained CZA exposure can drive high-level, treatment-emergent CZA resistance through a 45-nucleotide in-frame duplication within *bla*_KPC2_.

Persistent respiratory colonization despite CZA therapy likely amplified selective pressure. CZA has demonstrated reduced efficacy in treating pneumonia compared to other infection types (38, 39). Pneumonia and RRT are independent risk factors for CZA treatment failure and resistance emergence (40). When treating cIAIs, CZA was associated with inferior outcomes in patients with renal impairment (9). In this patient, the combination of cIAI, respiratory colonization, severe renal impairment on RRT, and immunosuppression created a high-risk scenario for CZA resistance emergence and poor outcome.

While KPC-44 serves as a particularly clear example of how mutations in the KPC enzyme can confer CZA resistance, numerous related adaptive variants have also been described (14, 15). These mutations—point substitutions, insertions, and deletions—cluster within three “hot spots” surrounding the β-lactamase active site (Fig. 4B). The 15-amino acid duplication defining KPC-44 occurs within the 270-loop motif. Structural and biochemical studies show that elongation of this loop increases local dynamic flexibility, perturbing neighboring Ω- and 240-loops (Fig. 4C). These conformational changes enhance ceftazidime hydrolysis while reducing avibactam inhibition, thereby promoting CZA resistance (41).

As this case highlights, KPC-producing *Enterobacterales* exposed to CZA selective pressure appear especially prone to *bla*_KPC_ mutation and treatment-emergent resistance. Coexistence of multiple β-lactamases was found to promote stepwise evolutionary trajectories toward BL/BLI resistance (42); in this case, additional β-lactamases (*bla*_SRT_, *bla*_TEM-1_, *bla*_SHV-30_, and *bla*_OXA-9_) may have contributed intermediate resistance, enabling selection of *bla*_KPC-44_. From the enzyme standpoint, the high intrinsic thermodynamic stability of KPC-2 likely allows it to tolerate destabilizing mutations, insertions, or deletions without loss of function (43). From the BL/BLI perspective, resistance to MVB and I-R typically results from reduced outer membrane permeability rather than novel *bla*_KPC_ variants (44). Collectively, these observations suggest that CZA-driven resistance likely arises from a complex interplay of organismal, enzymatic, and drug-specific factors.

Heteroresistance also likely contributed to the observed resistance evolution. This phenomenon—coexistence of bacterial subpopulations with differing genotypic and phenotypic resistance—has been increasingly recognized as a mechanism promoting antimicrobial resistance evolution and associated treatment failure (45). The distinct resistance and ARG profiles of *Sm*-4a and *Sm*-4b are consistent with such subpopulation dynamics, similar to findings in CZA-exposed KPC-producing *K. pneumoniae* (46). Overall, sequential isolates *Sm*-1 through *Sm*-4 demonstrate clonal evolution and emergence of heteroresistance in the *S. marcescens* lineage during dynamic antibiotic exposure.

CZA exposure in this case induced cross-resistance to both MVB and I-R. CZA resistance-conferring *bla*_KPC_ variants have not generally been linked with cross-resistance to other contemporary BL/BLI agents. For example, a prior report of *bla*_KPC-44_ in *K. pneumoniae* described high-level CZA resistance (MIC 128 µg/mL) while maintaining susceptibility to MVB and I-R (MIC ≤ 0.06 µg/mL for both) (47). In the *S. marcescens* isolates, the highest levels of BL/BLI resistance occurred in isolates carrying two *bla*_KPC-44_ copies on distinct plasmids (*Sm*-3b and *Sm*-4b), suggesting that both copy number and genetic context of the *bla*_KPC_ allele may influence the resistance phenotype. However, resistance determinants beyond *bla*_KPC-44_ likely also contribute to the broader BL/BLI resistance observed. In *K. pneumoniae*, MVB resistance often involves outer membrane porin mutations, occasionally conferring dual resistance to MVB and CZA (48, 49). Despite the evolution of broad BL/BLI resistance, the *S. marcescens* isolates remained susceptible to the siderophore cephalosporin cefiderocol, underscoring a preserved therapeutic option. Thus, while *bla*_KPC-44_ plays a central role, the expanded BL/BLI resistance phenotype likely reflects a multifactorial process involving both species- and strain-specific factors.

Previous studies have shown that the development of CZA resistance mediated by KPC Ω-loop variants in clinical isolates is often accompanied by restored carbapenem susceptibility (50–53). Subsequent *in vitro* meropenem exposure, however, can reselect carbapenem resistance while preserving CZA resistance (54). In contrast, the *S. marcescens* isolates in this case remained carbapenem resistant throughout sequential exposure to meropenem and then CZA (Fig. 2), despite biochemical evidence that KPC-44 has reduced hydrolytic activity against carbapenems relative to KPC-2 (41, 47). Although restoration of carbapenem susceptibility could theoretically mitigate the clinical impact of CZA resistance in a subset of KPC-producing CRE, this case exemplifies a scenario in which CZA-induced *bla*_KPC_ variation only expanded the resistance spectrum.

CZA remains an important therapeutic advance against KPC-producing CRE. However, this case highlights its vulnerability to treatment-emergent resistance selection and cross-resistance to multiple contemporary BL/BLI agents such as MVB and I-R. Preserving the efficacy of these agents will require a multifaceted approach integrating strategies to limit resistance selection—through antimicrobial stewardship, infection control practices, and optimized dosing regimens—along with continued efforts to elucidate the complex agent-, pathogen-, and host-specific mechanisms driving resistance evolution.

## Data Availability

All raw sequencing reads (Illumina short reads and Oxford Nanopore long reads) and assembled genomes generated in this study have been deposited in the National Center for Biotechnology Information (NCBI) under BioProject accession number PRJNA1345726.
